# Middle identification for rhesus monkeys is influenced by number but not extent

**DOI:** 10.1038/s41598-020-74533-8

**Published:** 2020-10-15

**Authors:** Rosa Rugani, Michael L. Platt, Zhaoying Chen, Elizabeth M. Brannon

**Affiliations:** 1 Department of General Psychology, University of Padua, via Venezia 8, 35100 Padua, PD Italy; 2 Department of Psychology, School of Arts and Sciences, University of Pennsylvania, Philadelphia, PA USA; 3 Department of Neuroscience, Perelman School of Medicine, University of Pennsylvania, Philadelphia, PA USA; 4 Marketing Department, The Wharton School, University of Pennsylvania, Philadelphia, PA USA

**Keywords:** Psychology, Zoology

## Abstract

Abstract concept learning provides a fundamental building block for many cognitive functions in humans. Here we address whether rhesus monkeys (*Macaca mulatta*) can learn the abstract concept of “middle” in a series of objects. First, we trained monkeys to select the middle dot in a horizontal series of three dots presented on a touchscreen. Monkeys maintained a preference to choose the middle dot despite changes in the appearance, location, and spacing of the horizontal series of dots. They maintained high performance when the color, shape and the length of the stimuli were new, indicating that their responses did not depend upon the particular appearance of the array items. Next, we asked whether monkeys would generalize the middle concept to a 7 dot series. Although accuracy decreased when the number of dots was increased, monkeys continued to preferentially select the middle dot. Our results demonstrate that rhesus macaques can learn to use a middle concept for a discrete set of items.

## Introduction

As animals navigate the physical world, spatial relationships and numerosity are two of the fundamental cues available to differentiate between similar objects. The nature of the representations underlying animal spatial cognition has long been the subject of intense debate. One common way to remember the position of a concealed goal is to memorize it relative to visual landmarks, which function as beacons^1^. A crucial issue is whether animals encode at least some of the geometric relationships among real-world objects^2^. This raises the question of whether animals are sensitive to abstract geometric relationships. Abstract geometric concepts depend upon relationships between stimuli and transcend the objects’ specific features. This question has been addressed with a hidden goal technique, which requires an animal to find a goal that bears a stable relationship to a set of reference points^3–7^. To determine which geometric relationships animals rely on, the position of the landmarks is then manipulated, in the so called “expansion test” paradigm^8,9^. For instance, one task that has been used to compare spatial abilities in different species, ranging from invertebrates^10^ to humans^6,7^, requires animals to localize the geometric center in the absence of beacons. Thus, the center identification requires the use of metric information extrapolated by spatial relations and distances between reference points. Honeybees learned to find a food source that stands in a constant spatial location from three identical landmarks in a triangular array. At test, the array was either contracted (by reducing the inter-landmarks distances) or expanded (by increasing the inter-landmarks distances). The bees searched closer when the array was contracted and farther away when the array was expanded^8^. These search patterns indicate that the honeybees flexibly adjusted their search distances from the landmarks to maintain a central position between them^10^. In other studies, pigeons and humans learned to search for a goal in the center of four landmarks that were arranged in a square on the floor or on a computer monitor^6,7^. When the array was expanded, pigeons searched at the same absolute distance, thus ignoring the geometrical center, while humans continued to search in the center^6,7^. Despite similar experiences, humans abstracted a geometric rule (see also^11^) whereas pigeons relied upon metric information. Similar to pigeons, gerbils^9^, squirrel monkeys^12^, marmosets^11^, orangutans^13^ and children (five-to-nine year-old^11^ and 4-to-10 year-old^13^) learned to locate a goal in the geometric center between shaped-organized landmarks. Subsequently, in an expansion test all 5 species continued to search at the previously-experienced distances from the landmarks and failed to integrate the relationships between the reference points to form an abstract concept of “center.”

Younger children (ages 3–5) tested in the same paradigm have yielded conflicting results. In one study they were not as a group able to learn the task, however children who did learn typically searched nearer the landmark but not in the center^11^. Another study created a more engaging version of the task in which children had to localize a hidden sensor to play music. In this test, children learned to identify the geometric center of different shapes and they also generalized to novel shapes and dimensions, suggesting engagement is important for learning and generalizing the concept of “center”^14^. Remarkably, day-old domestic chicks (*Gallus gallus*) were trained to identify the geometric center of one shape and transferred the rule to other shapes. Nevertheless in an expansion test, chicks searched both in the center and at the corresponding absolute distance from a wall^15–17^. This means that young birds relied on both absolute and relative metric information to guide search. Performance deteriorated when the task was conducted in circular arenas, suggesting that corners are essential landmarks that chicks use for abstracting the center principle^15^.

To address the same question of center identification, Kamil and Jones^2^ used a novel approach, which required Clark’s nutcrackers to extrapolate the point equidistant between two landmarks, thus removing confounds generated by the encoding of geometric configurations. Clark’s nutcrackers learned to find a seed buried equidistant between two landmarks. Across trials, the landmarks were set at five different distances, so that the absolute distance of the midpoint from each landmark varied during training. At test, when the landmark location varied the nutcrackers continued to search at the location equidistant between the landmarks. Similar results were obtained in another study with Clark's nutcrackers, jackdaws, and pigeons. All species learned the task, although nutcrackers showed more accurate responses. Nutcrackers and pigeons, but not jackdaws, succeeded in transferring the rule to novel distances^18^. Under similar training and experimental manipulations, capuchin monkeys^19^, bonobos^20^ and children of 4 and 5 years^21^ used both the geometric center and the absolute distance from the two landmarks.

Overall, these studies show that a variety of animal species proficiently use landmark-goal relations, and that, in some cases, they are able to represent abstract landmark-landmark relationships to locate a relational goal. All of these studies focused on the identification of the geometric center. By contrast, few studies have investigated the capability to identify the middle point in a discrete series of elements. To the best of our knowledge, the first empirical investigation of the concept of middle was performed in 1934, when Yerkes taught chimpanzees to identify the middle box in a row of three boxes for food reward. When presented with an increasing number of boxes (five, seven or nine), chimpanzees failed to generalize and choose the middle item in the series^22^. The failure with larger numbers of boxes has been attributed to the complexity of the experimental design, which required the animals to explore the inside of closed boxes. Using a simpler design, which used boxes that were less difficult to open, chimpanzees generalized to a row of five boxes^23^. Subsequent experiments showed that a single female chimpanzee could learn to identify the middle item in a half-circular arrangement of up to seventeen items, however there was no evidence of transfer from a smaller to larger numerosity^24,25^. This single chimpanzee also was successful when the objects were asymmetrically distributed across the overall configuration, suggesting she might have exploited a numerical strategy rather than a the concept of middle^26^. More recently, young domestic chicks were trained to peck the middle of a series of three beads and then generalized to preferentially pecking the middle bead in series of five, seven, and nine identical beads^27^.

**Figure 1:**
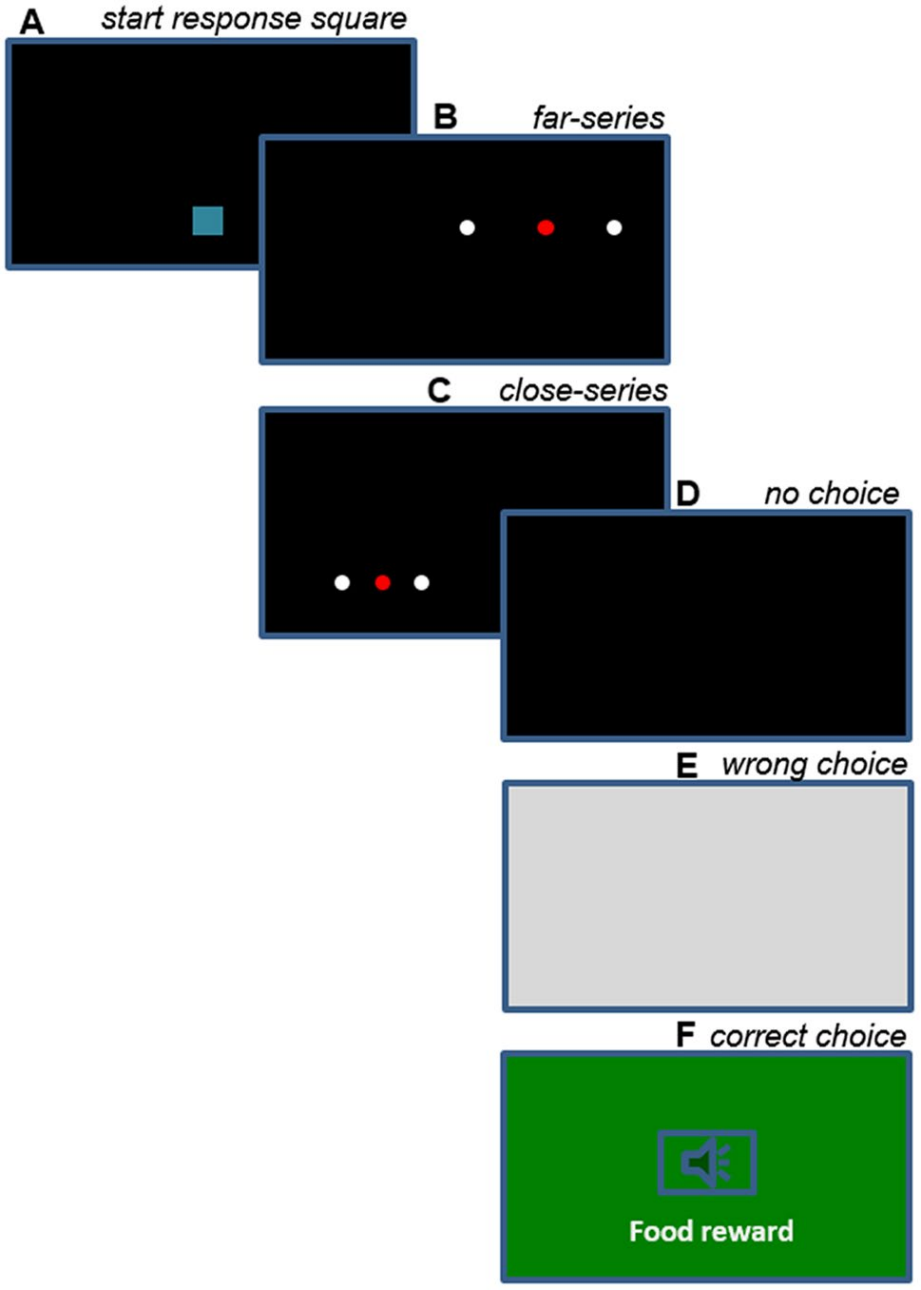
Schematic representation of the training procedure and of the training stimuli. Each trial began with the presentation of the catered start response square, (**A**) a stimulus appeared, in either its far or close version, (**B**,**C**) no choice within 5 s elicited a black screen, (**D**) choices of the lateral dots elicited a grey screen, (**E**) selection of the middle dot elicit a positive reward, a positive sound and a green screen (**F**).

Here we explore whether rhesus monkeys can use the abstract concept of middle to guide behavior, in a high-controlled computerized setting. We trained rhesus monkeys on a touch-screen task to select the middle dot in a series of three dots (Fig. 1). By using a touch-screen, we gained precise control over the appearance and spatial placement of stimuli, and eliminated odor cues or experimenter influence. Monkeys initiated each trial by touching a start response square that was always on the bottom central position. This way, before each trial, monkey attention was directed in the same area (Fig. 1A). On each trial, monkeys received a food reward if they selected the middle dot in a three-dot array. To avoid the possibility that monkeys responded by simply touching a specific position on the screen, we used two inter-dot distances (close inter-dot series: 0.75 cm and far inter-dot series: 2 cm) and 32 different absolute locations on the screen, balanced for left/right and up/down. During training the middle dot was illuminated red, while the lateral dots were illuminated white (Fig. 1B,C).

In two subsequent training sessions, we decreased the salience of the color cues by placing a red ring around the lateral white dots, which was respectively 0.25 cm 0.35 cm thick (Fig. 1B,b1,b2).

**Figure 2:**
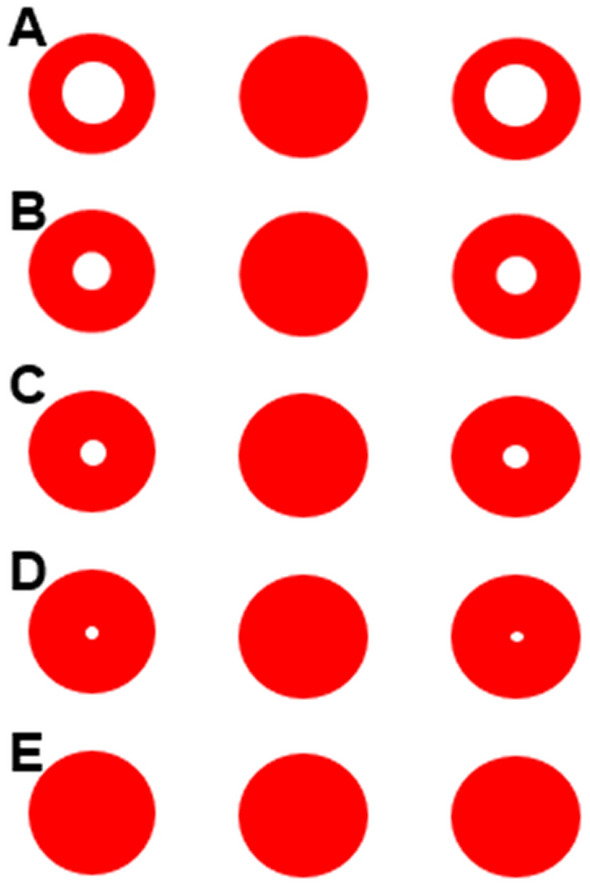
Schematic representation of the stimuli used in training 2 and 3 and in Experiment 1. (**A**) The stimuli used in training 2; (**B**) the stimuli used in training 3; (**C**) the stimuli used in Experiment 1 (**A**,**B**,**C**,**D**,**E**).

The first goal of this study was to understand how a visual color cue might aid identification of the middle item (Experiment 1). To address this question, we varied the thickness of the red ring around the lateral white dots, Fig. 2. We used three different kinds of stimuli: one previously experienced during training (ring thickness of 0.25 cm and 0.35 cm; Fig. 2A,B), which provided the color cue; a new one which provided a smaller color cue (ring thickness of 0.4 cm and 0.45 cm; Fig. 2C,D); and a completely novel one, which did not provide any color cue (the dots were all solid red, Fig. 2E). If monkeys relied solely on the color cue to identify the middle dot, their performance would decrease to chance when the dots were identical in color. We found that monkeys continued to respond to the middle dot with above chance accuracy when all three dots were solid red. Monkeys thus encoded and then proficiently used the sequential relationship between the dots to detect the middle.

**Figure 3:**
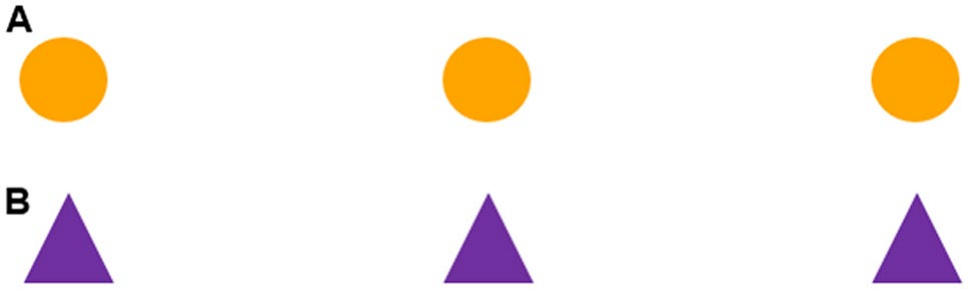
The stimuli used in the spatial expanded test, Experiment 2. (**A**) Stimuli used in the ‘Color and distance’ condition; (**B**) stimuli used in the ‘Shape, color and distance’ condition.

The gold standard for abstract-concept learning is transfer to novel stimuli. We designed two new probe conditions to assess whether the rule the monkeys learned would transfer to physically distinct elements, characterized by new items and new inter-dots distances (Experiment 2, Fig. 3).

To this aim we used three different items: red dots, orange dots (Fig. 3A) and purple triangles (Fig. 3B). Each stimulus comprised three identical items and was presented in multiple location on the screen. The inter-item distance was always new: 1 cm for red dots and 3 cm for orange dots and purple triangles. The increased distance on probe trials was specifically used to obtain a spatial expansion test, which prevent monkeys to rely on an absolute distance from either end of the array to solve the task. This way we created two probe conditions: the ‘Color and distance’ condition (Fig. 3A), and the ‘Shape, color and distance’ condition (Fig. 3B). Monkeys chose only the middle item significantly above chance, also when the stimuli were new for distance, color and shape, demonstrating that they can apply the learned rule to new items and new array’s lengths. Nevertheless this test does not allow us to disentangle if monkeys relied on a middle strategy or on an absolute numerical/ordinal strategy, since the middle item was also the second from the left and right side. For this reason we increased the number of items to seven, in Experiment 3.

**Figure 4:**
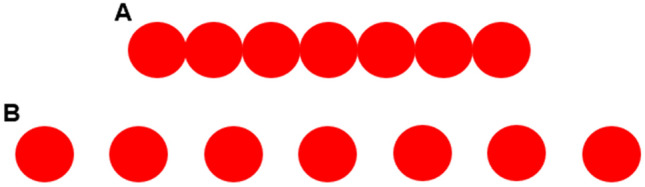
The stimuli used in the numerical expanded test, Experiment 3. (**A**) The close 7-dot series; (**B**) the far 7-dot series.

The aim of Experiment 3 was to test whether monkeys can generalize the middle concept to an expanded series of seven dots (Fig. 4).

We used two inter-dot distances (close 7-dot series and far 7-dot series) and multiple absolute locations on the screen. To avoid extinction, 90% of trials contained 3-dots and were differentially reinforced, whereas 10% of trials contained 7-dots and were non-differentially reinforced. We found that monkeys selected the middle position with above chance accuracy on the 7-dot probe trials, indicating they can extrapolate sequential relationships and can flexibly use them to navigate novel and larger series. Monkeys were more accurate in responding to the 3-dot series than to the 7-dot series. Thus, monkeys learned an abstract concept of middle and the number of elements, but not the overall length of the series, shaped their responding. Overall our findings demonstrate that monkeys can spontaneously extrapolate geometric relationships and flexibly use the middle concept.

## Results

### Experiment 1

**Figure 5:**
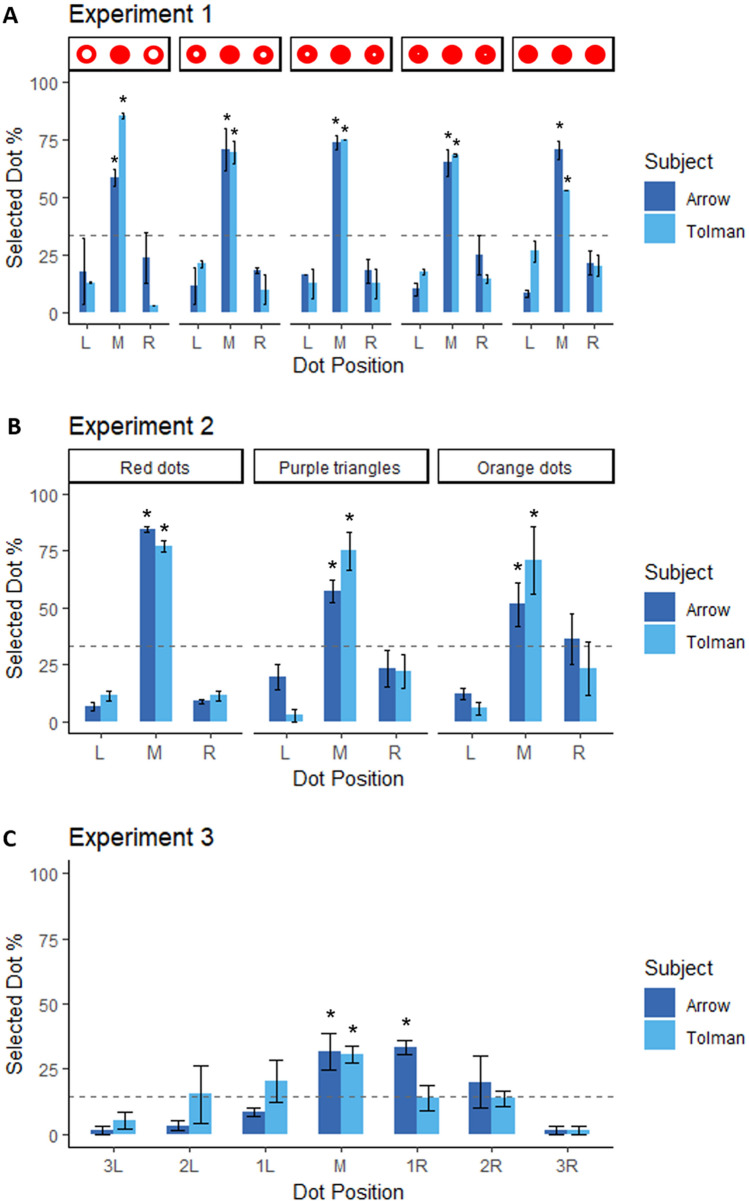
Results of the Experiment 1 (**A**), Experiment 2 (**B**) and Experiment 3 (**C**). Monkeys preferentially selected the middle dot in both 3 and 7 dot series. Error bars = SEM. Dotted line shows chance performance (3-dots series, Y = 33.333; 7-dot series, Y = 14.286).

Monkeys responded similarly on close and far 3-dot series. Therefore, we combined the results for these two trial types throughout the remainder of our analyses. We used one-tailed exact binomial tests to determine whether the middle position was selected with above chance expectation on each trial type. On all trial types, monkeys selected the middle dot with above chance accuracy (see Table 1, Fig. 5A). Moreover, accuracy did not differ across the five trial types used in Experiment 1 (Arrow: X^2^ = 4.498, df = 4, p = 0.343, Phi = 0.143; Tolman: X^2^ = 1.686, df = 4, p = 0.793, Phi = 0.091). Despite the availability of the white color cue, monkeys spontaneously encoded and used the sequential relationships to detect the middle element.

**Table 1:** Results for different stimulus types in Experiment 1 for each monkey.

	Difference between the inter-dot distance in each series				Choice for the middle dot			
	X^2^	df	p	Phi	Correct trials	Valid trials	p	Cohen’s d
**Arrow**								
*Solid red*	*1.222*	*2*	*0.543*	*0.138*	*34*	*64*	< *0.001*	*0.403*
*Ring* = *0.45 cm*	*0.209*	*2*	*0.900*	*0.057*	*43*	*63*	< *0.001*	*0.683*
*Ring* = *0.4 cm*	*4*	*2*	*0.135*	*0.25*	*48*	*62*	< *0.001*	*0.921*
Ring = 0.35 cm	2.053	2	0.228	0.218	43	62	< 0.001	0.738
Ring = 0.25 cm	0.955	2	0.620	0.124	53	62	< 0.001	1.130
**Tolman**								
*Solid red*	*1.086*	*2*	*0.581*	*0.133*	*43*	*61*	< *0.001*	*0.763*
*Ring* = *0.45 cm*	*2.493*	*2*	*0.288*	*0.207*	*38*	*58*	< *0.001*	*0.656*
*Ring* = *0.4 cm*	*5.826*	*2*	*0.054*	*0.309*	*45*	*61*	< *0.001*	*0.836*
Ring = 0.35 cm	4.229	2	0.121	0.263	43	61	< 0.001	0.763
Ring = 0.25 cm	9.908	2	0.007	0.406	35	60	< 0.001	0.508

New stimuli which were not experienced during training are indicated in italics.

### Experiment 2

#### Training trials

Both monkeys responded differently to the three red dots (Arrow: X^2^ = 103.98, df = 2, p < 0.001, Phi = 1.081; Tolman: X^2^ = 76.455, df = 2, p < 0.001, Phi = 0,932; Chi-squared for given probabilities test). They continued to select only the middle dot with above chance accuracy (Arrow: number of successes = 75, number of trials = 89, p < 0.001, Cohen’s d = 1.096; Tolman: number of successes = 68, number of trials = 88, p < 0.001, Cohen’s d = 0.917; Exact binomial test), while the other dots were selected at or below chance expectation.

#### Test trials

The monkeys responded differently to the three items in the ‘Color and distance’ condition (Arrow: X^2^ = 7.938, df = 2, p = 0.019, phi = 0.498; Tolman: X^2^ = 24.4, df = 2, p < 0.001, phi = 0.835; Chi-squared test). They chose only the middle item with above chance expectation (Arrow: number of successes = 17, number of trials = 32, p = 0.016, Cohen’s d = 0.403; Tolman: number of successes = 25, number of trials = 35, p < 0.001, Cohen’s d = 0.783; Exact binomial test, Fig. 5B).

The monkeys responded differently to the three items in the ‘Shape, color and distance’ condition (Arrow: X^2^ = 8.971, df = 2, p = 0.011, phi = 0.506; Tolman: X^2^ = 30.167, df = 2, p < 0.001, phi = 0.915; Chi-squared test). They chose only the middle triangle above chance expectations (Arrow: number of successes = 20, number of trials = 35, p = 0.003, Cohen’s d = 0.484; Tolman: number of successes = 27, number of trials = 36, p < 0.001, Cohen’s d = 0.864; Exact binomial test).

### Experiment 3

#### Three dot training trials

We first examined whether monkeys responded differently in the differentially rewarded close and far 3-dot series. Arrow responded identically in the close and far-dot series (X^2^ = 5.910, df = 2, p = 0.052, Phi = 0.097; Pearson’s Chi-squared test), whereas Tolman’s responses differed for the close and far-dot series (X^2^ = 23.723, df = 2, p-value < 0.001, Phi = 0.196; Pearson’s Chi-squared test). Nevertheless, both monkeys continued to select the middle dot with above chance accuracy for both close and far trials (Arrow: close-series: number of successes = 179, number of trials = 294, p < 0.001, Cohen’s d = 0.560; far-series: number of successes = 228, number of trials = 337, p < 0.001, Cohen’s d = 0.701; Tolman: close-series: number of successes = 213, number of trials = 313, p < 0.001, Cohen’s d = 0.710; far-series: number of successes = 193, number of trials = 305, p < 0.001, Cohen’s d = 0.906; Exact binomial test), while the other dots were selected at or below chance expectation.

#### Seven dot test trials

Monkeys exhibited similar accuracy for selecting the middle dot on close and far trials (Arrow: X-squared = 0.053, df = 1, p-value = 0.819, phi = 0.053; Tolman: X^2^ = 0.222, df = 1, p-value = 0.637, phi = 0.111; Pearson’s Chi-squared test), leading us to combine trial types for subsequent analyses. As shown in Fig. 5c the monkeys responded differently to the seven dots (Arrow: X^2^ = 40.2, df = 6, p < 0.001, phi = 0.906; Tolman: X^2^ = 22.508, df = 6, p < 0.001, phi = 0.618; Chi-squared for given probabilities test). Both monkeys chose the middle dot above the 14.286% chance expectation (Arrow: number of successes = 19, number of trials = 60, p < 0.001, Cohen’s d = 0.420; Tolman: number of successes = 18, number of trials = 59, p < 0.001, Cohen’s d = 0.39; Exact binomial test, Fig. 5C). Arrow also touched the dot adjacent and to the right of the central dot more frequently than expected by chance (p-value < 0.001, Cohen’s d = 0.464). Notably, neither monkey responded preferentially to the second element from the left nor the second element from the right as would be expected if they had extrapolated an absolute numerical rule from the three-dot training.

To test whether monkeys used a numerical middle rule or a spatial middle rule we attempted to compare accuracy on trials with the same physical extent and different numerosity. If monkeys used a spatial middle rule we would expect similar accuracy for sequences with the same physical extent. In contrast, if monkeys used a numerical middle rule accuracy should decrease with increasing numerosity as predicted by Weber’s law. For each experimental session and for each series, accuracy was computed as the number of correct trials/total number of valid trials × 100. Despite equal spatial extent, accuracy was much higher for the far 3-dot trials compared to the close 7-dot trials (Arrow: three-far: Mean = 67.545, SE = 3.989; seven-close: Mean = 30.000, SE = 9.229; V = 50, p = 0.019, r = 0.522; Tolman: three-far: Mean = 63.118, SE = 3.379; seven-close: Mean = 28.333, SE = 5.000; V = 55, p = 0.002, r = 0.692; Paired Wilcoxon test). However, one might argue this is not a fair comparison given that the 3-dot trials were reinforced and the 7-dot trials were not. We therefore compared accuracy on the three-orange dots and three-purple triangles from Experiment 2 and the far-7 dots from Experiment 3, which similar but not identical lengths (respectively 13 cm and 11.5 cm). Once again, accuracy was greater on 3-item trials (Arrow: three-items: Mean = 54.293, SE = 5.047; seven-items: Mean = 33.333, SE = 8.607; V = 1 , p = 0.031 , r = 0.538; Tolman: three-items: Mean = 72.980, SE = 7.557; seven-items: Mean = 33.333, SE = 9.938; V = 1 , p = 0.053, r = 0.484; Paired Wilcoxon test). Collectively these analyses suggest that monkeys used a numerical middle rule rather than a spatial middle rule.

## Discussion

We investigated whether rhesus monkeys can represent the abstract “middle” concept. We first trained monkeys to select the middle position in a horizontal array of three items. During training we gave the monkeys a clear visual color cue that differentiated the middle item from the other items. Monkeys spontaneously extracted more abstract relational information such that they maintained high performance when the physical differences between the lateral and middle item were eliminated. Monkeys further ignored the physical distance between the elements and the absolute location of the elements on the screen.

Additional transfer conditions revealed that monkeys used a relative, rather than absolute numerical middle rule. They generalized the rule to three item sequences that were both spread out and new in color and shape. Monkeys also generalized the rule to sequences of seven items that were not differentially reinforced. Their performance was again insensitive to variations in inter-element spacing and to the absolute position of the array on the screen, showing that they did not rely on spatial cues. Monkeys did not use specific metric information to detect the middle, but instead flexibly applied the numerical middle rule to novel and expanded series. This demonstrates that monkeys extracted the middle item in a series of identical items, providing the first evidence of middle abstraction in a discrete series of sequential and discrete elements. Our results align with previous studies showing various animals can extract the central geometric point in a shape (honeybees^8^, nutcrackers^2^, pigeons^18^) and with other studies showing some animals can reliably learn to identify the middle object in a series (domestic chicks^27^). We speculate that our training procedure, which used two different inter-item distances and varied the location of the sequence on the screen, encouraged monkeys to learn and apply the abstract numerical middle rule rather than rely on spatial relationships. This explanation is consistent with a prior study in which birds learned to identify a point equidistant between two landmarks. As in our study, nutcrackers learned to find the equidistant point between two landmarks, when multiple inter-landmark distances were used during training^2^, but relied on absolute metric distance when only a single inter-landmark distance was used during training^28^.

Although our intention was that monkeys would learn a numerical middle concept, an alternative strategy would have been to learn an absolute numerical rule such as “choose the second dot from the left” or “choose the second dot from the right”. Previous studies have shown that monkeys^29,30^, and other animals (rats^31,32^, domestic chicks^33,34^, Clark’s nutcrackers^35^, fish^36,37^ and bees^38^) can learn this type of absolute numerical rule. However, our data clearly show that monkeys did not preferentially choose the second from the left or the right in the 7-dot sequence. A second question is whether monkeys learned a middle concept based on number, whereby they estimated the number of dots from each end of the series, or a relative spatial strategy, whereby they estimated the geometric midpoint from each end of the sequence. While our results do not definitively differentiate between these two strategies they are more consistent with the numerical middle rule. Although the far 3-dot trials and the close seven-dot trials had the same spatial extent, accuracy was higher on the 3 dot trials suggesting that the middle concept was based on discrete items. Future studies should more systematically investigate the relative role of spatial and numerical cues in abstracting a middle rule. For example, an interesting question is whether monkeys could transfer between a numerical and spatial middle concept by training with discrete sequences and testing with continuous lines. While many questions remain, our findings demonstrate that rhesus monkeys can learn an abstract middle concept and apply it to sequences that vary in color, shape, length and numerosity.

## Materials and methods

All procedures reported in this study were approved by the Institutional Animal Care and Use Committee (IACUC) of the University of Pennsylvania, and performed in accordance with their relevant guidelines and regulations. The PROTOCOL # 806050 have been also reviewed by the Institutional Review Board (IRB) using the expedited procedure set forth in 45 CFR 46.110 and approved on 05-Feb-2019.

### Subjects

Two male rhesus macaques (*Macaca mulatta)*, named Arrow (5 year old) and Tolman (6 year old), served as subjects. Monkeys were housed in pairs in a vivarium and were separated for testing. Fresh fruit and vegetables and LabDiet’s Monkey Diet biscuits were provided daily. Water was available *at libitum*. For both monkeys this was their first touch screen study.

### Apparatus and stimuli

Experimental apparatus consisted of a 15-in touch-sensitive computer monitor (Elo TouchSystems, Menlo Park, CA) and a food pellet reward delivery system (Med Associates, St. Albans, VT). These were attached to the front of the macaque’s home cage. Stimulus presentation, reward delivery and data collection were conducted on PsychoPy3.

The training and testing stimuli consisted of red or white dots on a black background, these were also created in PsychoPy3^39^. The dimension of the dots and the numbers of dots displayed depended on the experimental phases.

### Shaping

#### Shaping 1

Monkeys’ behavior was first shaped to touch a single red (RGB = 255, 0, 0) dot, randomly presented on the left/right, up/down portion of the monitor. The background was black (as in all the following shaping, training and testing phases). Since it was the first touch screen experience for both subjects, the diameter of the dot was large (16 cm) and was progressively halved to until it was 1 cm. During shaping the response time was unlimited and whenever they touched the dot, they received a food pellet reward, a pleasant sound, and a green screen. We then inserted a start response—a 2 × 2 cm blue square. The start response square was located at the bottom-center of the screen (located at 0, − 8) on every trial to anchor monkeys’ attention immediately before each trial. Immediately after the start response was made a 1 cm red dot appeared in one of 32 different screen locations. We used a virtual Cartesian coordinate system with one centimeter as the standard unit. The origin of the coordinate is the center of the screen (0, 0; which respectively indicate the position on the X and Y axis). The position of the dot was determined by the position of its center on the two diagonals, with the absolute values of coordinates ranging from 1 to 8. This way, the dot appeared in 8 different locations on each quadrant, moreover the position of the dots in each quadrant was symmetrically displaced on the horizontal and vertical axis.

#### Shaping 2

Once the monkeys reliably responded to the start stimulus and the red dot that appeared in any of 32 locations a response was required within 5 s to elicit a reward. If a monkey failed to respond within 5 s this caused a 5-s dark screen and no food reward. Session length was then increased to 64 trials and a performance criterion of 70% accuracy over two consecutive sessions was required to advance to 3-dot training.

### 3-dot training

#### Training 1

Training sessions consisted of 64 trials, designed to train the monkeys to respond to the middle dot in a series of three horizontally aligned dots. Each dot was 1 cm in diameter and the dots were presented 2 cm or 0.75 cm apart (these were respectively called far- and close-series). The absolute position of each series was determined by the position of the central dot, as described in Shaping 2. In Training phase 1 the central dot was red, while the two lateral ones were solid white. An array of three dots was presented immediately after each start response. In the first phase of training only correct responses were recorded and reinforced, while incorrect responses did not end the trial. A lack of response within 5 s elicited a 5-s timeout and no reward.

#### Training 2

The procedure was similar to Training 1, but here we differentiated between correct and incorrect responses. In this phase incorrect responses ended the trial and were followed by a 5-s grey screen and no reward.

#### Training 3

In this phase we decreased the color cue by placing a red ring around the lateral white dots. In two consecutive trainings ring thickness was 0.025 cm and 0.035 cm.

#### Power analysis

We conducted a power analysis for binomial distribution using the library(pwr) in R, to determine how many trials to run in each experiment. Sample size was calculated for a power of 0.08, a 0.05 one-sided (alternative = “greater”) significance level, and medium/small effect size (0.45). The outcome was 30.531. To balance the left/right and up/down position of each series in Experiment 1, we included 32 trials for each (far and close) of the five trial types. In Experiment 2, we ran 30 trials for each (far and close) 7-dot series.

#### Experiment 1

To test whether monkeys were using the color cue to identify the middle dot we systematically varied the thickness of the red ring around the lateral white dots (0.25 cm, 0.35 cm, 0.4 cm, 0.45 cm). Thus two of the 5 trial types were identical to Training phase 3 (ring thickness of 0.25 cm, 0.35 cm); two trial types were new but contained the white cue (ring thickness of 0.4 cm and 0.45 cm); the final trial type was new and lacked the color cue. For each trial type we used the two inter-dot distances and the 32 absolute locations as described in Training 1. Each session consisted of 64 trials. We collected 64 trials for each of the five trial types (320 trials overall) and used only one trial type per session.

#### Training 4

We increased the proportion of trials in which all three dots were red over 5 phases (Phase 1: 20%; Phase 2: 40%; Phase 3: 60%; Phase 4: 80%; Phase 5: 100%). Moving from a phase to the subsequent one we replaced the trials characterized by the larger white cue with the complete red one.

Expansion experiments: Monkeys underwent two expansion experiments: a spatial expansion experiment (Experiment 2) and a numerical/spatial expansion experiment (experiment 3). In each experimental session, the novel test trials were intermixed with familiar training trials. In the training trials three red dots (diameter = 1 cm) were used as stimulus and were differentially reinforced. In both experiments the two novel test trials were non-differentially reinforced such that any choice resulted in reward. To balance the position on horizontal and vertical axes, the position of the probe series were always centered on the horizontal axis (x = 0); the position on the vertical axis could be 0, − 3 or + 3; the position of the training series varied as described for Training 1.

#### Experiment 2

Monkeys were presented with new items, spaced 5 cm apart one another. A stimulus was composed of three orange dots (RGB = 255, 165, 0; diameter = 1 cm), and the other was composed of three purple isosceles triangles (RGB = 128, 0, 128; height = 1 cm; width = 1 cm). Sessions consisted of 56 trials total: the training and test trials were randomly intermixed: 12 test trials (for each stimulus, and four for each height on the vertical axis).

#### Experiment 3

Monkeys were tested with two novel trial types to assess the generality of the middle rule that the monkeys had learned. Both trial types had seven solid red dots and differed only in inter-dot distances (0 and 0.75 cm). Sessions consisted of 70 trials total: the training and test trials were randomly intermixed: 64 training trials and 6 test trials (3 for each condition, and one for each height on the vertical axis).

## Data Availability

Our datasets Data are stored and backed up on the Research Data Unipd server. The Research Data Unipd is Research Data Repository that meets the demands of a FAIR data storage (Findable, Accessible, Interoperable, Reusable) in accordance with the Guidelines on FAIR Data Management in Horizon 2020–July 2016; https://researchdata.cab.unipd.it/id/eprint/372.
